# Autoantibodies neutralizing type I interferons in 20% of COVID-19 deaths in a French hospital

**DOI:** 10.21203/rs.3.rs-915062/v1

**Published:** 2021-10-01

**Authors:** Angélique Chauvineau-Grenier, Paul Bastard, Antoine Servajean, Adrian Gervais, Jérémie Rosain, Emmanuelle Jouanguy, Aurélie Cobat, Jean-Laurent Casanova, Benjamin Rossi

**Affiliations:** 1 Medical Biology Department, Robert Ballanger Hospital, Aulnay-Sous-Bois, France; 2 Laboratory of Human Genetics of Infectious Diseases, Necker Branch, INSERM U1163, Necker Hospital for Sick Children, Paris, France.; 3 University of Paris, Imagine Institute, Paris, France.; 4 St. Giles Laboratory of Human Genetics of Infectious Diseases, Rockefeller Branch, The Rockefeller University, New York, NY, USA.; 5 University of Paris, UFR de Médecine, site Xavier Bichat, Paris, France; 6 Howard Hughes Medical Institute, New York, NY, USA.; 7 Department of Internal Medicine, Robert Ballanger Hospital, Aulnay-Sous-Bois, France

**Keywords:** Autoantibodies, Type I interferons, COVID-19, mortality

## Abstract

Recent studies reported the presence of pre-existing autoantibodies (auto-Abs) neutralizing type I interferons (IFNs) in at least 15% of patients with critical or severe COVID-19 pneumonia. In one study, these auto-Abs were found in almost 20% of deceased patients across all ages. We aimed to assess the prevalence and clinical impact of the auto-Abs to type I IFNs in Seine-Saint-Denis district, which was one of the most affected areas by COVID-19 in France during the first wave.

We tested for the presence of auto-Abs neutralizing type I IFNs in a cohort of patients admitted for critical COVID-19 pneumonia during the first wave in the spring of 2020 in medicine departments at Robert Ballanger Hospital, Aulnay sous Bois.

We found circulating auto-Abs that neutralized 100 pg/mL IFN-α2 and/or IFN-ω in plasma 1/10 in 7.9% (11 of 139) of patients hospitalized for critical COVID-19. The presence of neutralizing auto-Abs was associated with an increased risk of mortality as these auto-Abs were detected in 21% of patients who died from COVID-19 pneumonia. Deceased patients with and without auto-Abs did not present overt clinical differences. These results confirm both the importance of IFN-I immunity in host defense against SARS-CoV-2 infection and the usefulness of detection of auto-Abs neutralizing type I IFNs in the management of patients.

## Introduction

Since the onset of the COVID-19 pandemic in December 2019, at least 220 million people have been infected, and most likely many more. Nevertheless, only about 10% of these individuals developed hypoxemic COVID-19 pneumonia (severe, or critical in about 3% of cases). There have been at least 4 million deaths, and most likely closer to 7–9 million. The clinical spectrum of SARS-CoV-2 infection is therefore vast, ranging from silent infection to lethal disease. A few epidemiological risk factors have been identified. The most important one is age, with a risk of life-threatening disease doubling every five years. Gender as well as several others risk factors have been described (*e.g* obesity) but with relatively modest effects [1,2]. In each demographic category, however, there remains vast inter-individual clinical variability.

Recent studies showed the important role of type I Interferons (IFNs) in protective immunity against SARS-CoV-2. Inborn errors of type I IFNs immunity were described in patients with life threatening COVID-19. Rare inborn errors of autosomal genes controlling Toll-like receptor 3 (TLR3) and interferon regulatory factor 7 (IRF7)- dependent type I IFNs immunity were initially described [3] and more recently X-linked recessive TLR7 deficiency [4]. These inbor errors impair the production or amplification of type I IFNs in response to SARS-CoV-2. Deficiency of the TLR3 pathway incriminated pulmonary epithelial cells, while that of the TLR7 pathway incriminated plasmacytoid dendritic cells.

Interestingly, several studies showed that at least 10% of patients with life threatening (critical) COVID-19 pneumonia presented autoantibodies (auto-Abs) neutralizing type I IFNs, mostly the thirteen individual IFN-α and IFN-ω [5]. These auto-Abs were present before infection by SARS-CoV-2 in the patients tested and in 0.33% of uninfected controls from before the pandemic. They were not detected in patients with asymptomatic or mild SARS-CoV-2 infection. They were also shown to block the anti viral activity of correspondent type I IFNs against SARS-CoV-2 *in vitro* and *in viv*o[5].These findings were replicated in other cohorts in Amsterdam, Madrid, San Francisco, Lyon and New Haven[6–13]

Recently, auto-Abs neutralizing lower, more physiological concentrations of type I IFNs were detected in 15 to 20% of patients with critical COVID-19, notably in more than 20% of critical patients over 80 years of age. When also considering auto-Abs to IFN-beta, they were present in about 20% of deceased patients across all ages [14]. Surprisingly, the study of more than 34,000 individuals showed that the prevalence of these auto-Abs in the general population increases with age, notably after the age of 70 (4%), providing an explanation for the increased risk of severe COVID-19 with age [14].

In this context, we aimed to assess the prevalence and clinical impact of the auto-Abs to type I IFNs in Seine-Saint-Denis department, which was one of the most affected areas by COVID-19 in France during the first wave of the pandemic[15]. We thus tested for the presence of auto-Abs to type I IFNs neutralizing different doses in a cohort of patients admitted for critical COVID-19 pneumonia during the first wave in the spring of 2020. Moreover, a commercial ELISA kit for the determination of anti–IFN-α auto-Abs was also evaluated.

## Materials and Methods

### Study population

A cohort of 246 patients admitted for critical COVID 19 pneumonia was constituted in Robert Ballanger Hospital, Aulnay sous Bois, France, during the first wave of the pandemic in order to assess factors associated with clinical outcomes in patients hospitalized for Covid-19 [16]. This study was approved by a research ethics committee and was registered on clinicaltrials.gov (NCT04366206).

Included patients were hospitalized in medicine departments dedicated to treat COVID-19 patients and severity criteria required a pulse oxygen saturation (SpO2) ≤ 96% despite oxygen support ≥ 6 L/min with oxygen mask, for more than 6 h. All patients therefore had ‘critical’ COVID-19 pneumonia [17]. As this cohort was constituted to assess associations between treatments and outcomes like need of ventilation mechanic and mortality, patients with invasive mechanical ventilation and those in Intensive Care Unit (ICU) before the admission in medecine were excluded..

From this cohort of 246 patients, 139 patients were retrospectively selected because of an available serum sample that was collected during the acute phase of disease and stored in the laboratory sample collection. For some of them, serum sample was also collected about one year later infection.

### Detection of anti-cytokine auto-Abs by Gyros and ELISA

Biological serum samples were analyzed for the determination of anti–IFN-α and anti–IFN-ω auto-Abs by Gyros technology as previously described [14].

Cytokines, recombinant human (rh)IFN-α2 (Milteny Biotec, ref. number 130-108-984) or rhIFN-ω (Merck, ref. number SRP3061), were first biotinylated with EZ-Link Sulfo-NHS-LC-Biotin (Thermo Fisher Scientific, cat. number A39257), according to the manufacturer’s instructions, with a biotin-to-protein molar ratio of 1:12. The detection reagent contained a secondary antibody, Alexa Fluor 647 goat anti-human IgG (Thermo Fisher Scientific, ref. number A21445) diluted in Rexxip F (Gyros Protein Technologies, ref. number P0004825; 1/500 dilution of the 2 mg/mL stock to yield a final concentration of 4 μg/mL). Buffer PBS-T 0.01% and Gyros Wash buffer (Gyros Protein Technologies, ref. number P0020087) were prepared according to the manufacturer’s instructions. Plasma or serum samples were then diluted 1/100 in PBS-T 0.01% and tested with the Bioaffy 1000 CD (Gyros Protein Technologies, ref. number P0004253), and the Gyrolab X-Pand (Gyros Protein Technologies, ref. number P0020520). Cleaning cycles were performed in 20% ethanol.

Moreover a commercially available ELISA kit (Human Anti-IFN alpha ELISA Kit, Thermo Fisher Scientific, ref number BMS217, hereafter referred to as “ELISA”) was also used for the quantitative detection of human anti-IFN α auto-Abs. In brief, microwells, that were coated with recombinant human IFN-α, were incubated with 1:5 dilutions of serum samples from the patients for 2 h at room temperature. Plates were thoroughly washed. Horseradish peroxidase (HRP)–conjugated human IFN-α protein was added and plates were incubated for 1 h at room temperature and washed. Substrate solution reactive with HRP was added and a colored product was formed in proportion to the amount of human anti-IFNα present in the sample or standard. The reaction was terminated by addition of acid and absorbance was measured at 450 nm.

### Functional evaluation of anti-cytokine auto-Abs

The blocking activity of anti-IFN-α2, anti-IFN-ω and anti-IFN-β auto-Abs was determined with a reporter luciferase activity as previously described [14].

Briefly, HEK293T cells were transfected with a plasmid containing the *Firefly* luciferase gene under the control of the human *ISRE* promoter in the pGL4.45 backbone, and a plasmid constitutively expressing *Renilla* luciferase for normalization (pRL-SV40). Cells were transfected in the presence of the X-tremeGene9 transfection reagent (Sigma-Aldrich, ref. number 6365779001) for 24 hours. Cells in Dulbecco’s modified Eagle medium (DMEM, Thermo Fisher Scientific) supplemented with 2% fetal calf serum (FCS) and 10% healthy control or patient serum/plasma (after inactivation at 56°C, for 20 minutes) were either left unstimulated or were stimulated with IFN-α2 (Milteny Biotec, ref. number 130-108-984), IFN-ω (Merck, ref. number SRP3061), at 10ng/mL or 100pg/mL, or IFN-β (Milteny Biotech, ref. number: 130-107-888) at 10ng/mL, for 16 hours at 37°C. Each sample was tested once for each cytokine and dose. Finally, cells were lysed for 20 minutes at room temperature and luciferase levels were measured with the Dual-Luciferase® Reporter 1000 assay system (Promega, ref. number E1980), according to the manufacturer’s protocol. Luminescence intensity was measured with a VICTOR-X Multilabel Plate Reader (PerkinElmer Life Sciences, USA). *Firefly* luciferase activity values were normalized against *Renilla* luciferase activity values. These values were then normalized against the median induction level for non-neutralizing samples, and expressed as a percentage. Samples were considered neutralizing if luciferase induction, normalized against *Renilla* luciferase activity, was below 15% of the median values for controls tested the same day.

### Statistical Analyses

To compare clinic characteristics, continuous variables are shown with the median and Standard Deviation (SD) and dichotomous variables are presented with the number of events and percentages.

Given the small samples size, a Fisher test was used to analyze the effect of dichotomous variables and a Mann-Whitney test for continuous variables.

## Results

### Detection of auto-Abs neutralizing IFN-α2 and/or IFN-ω

We first assessed the circulating levels of auto-Abs against IFN-α2 and IFN-ω using the Gyros technology in 139 patients with critical COVID-19 pneumonia. We found that 107 (77%) patients with critical COVID-19 have auto-Abs against IFN-α and/or IFN-ω by Gyros technology. Intermediate titer (between 30 and 100, as previously described [14]) were frequent, as found in 66.9% (93 of 139) of patients with critical COVID-19 and high levels, above 100, were found in 10.1% (14 of 139) patients with critical COVID-19.

We then tested the neutralizing activity of all these samples against IFN-α and IFN-ω *in vitro* at high concentrations of type I IFNs (10ng/ml) and lower concentrations (100pg/ml) whether they display high titer by Gyros assay or not. We found that 2.9% (4 of 139) of patients had auto-Abs neutralizing 10 ng/ml of IFN-α2 and/or IFN-ω. Three of these patients had auto-Abs neutralizing high concentrations of IFN-α2 and IFN-ω and 1 patient had only auto-Abs neutralizing high concentrations of IFN-α2. Moreover, some patients presented auto-Abs neutralizing only 100 pg/ml of IFN-α2 and/or IFN-ω but not higher concentrations. We found that 5% (7 of 139) of patients had auto-Abs neutralizing only lower concentrations of IFN-α2 or IFN-ω. Two of these patients had auto-Abs against only IFN-α2 and 5 patients had auto-Abs against IFN-ω only.

**Fig 1:**
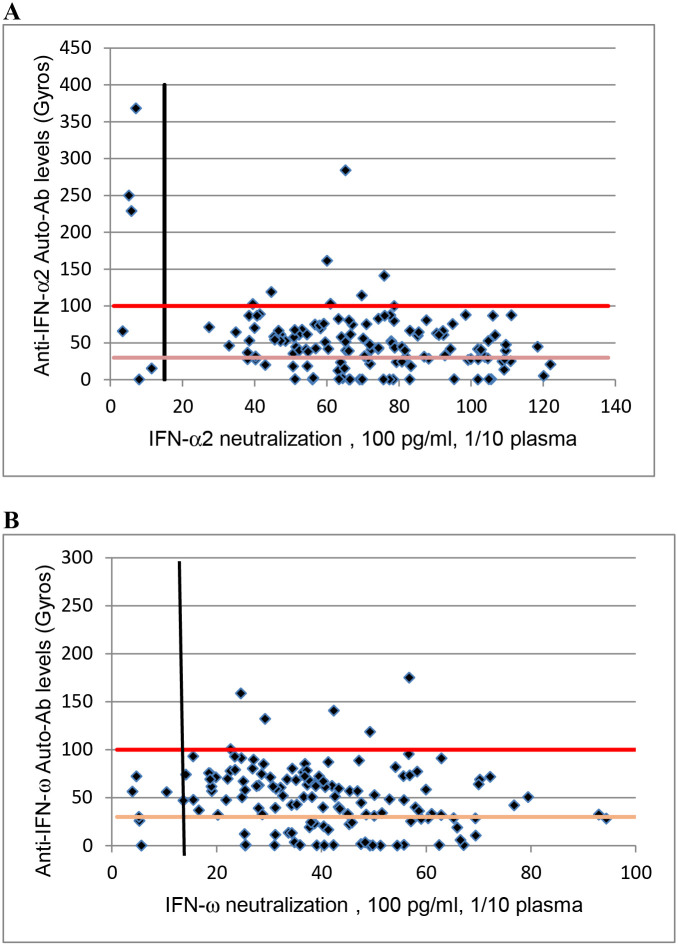
**A**: Plot of anti–IFN-α2 auto-Abs levels, as determined by Gyros, against their neutralization capacity at 100 pg/ml. The vertical dotted line indicates neutralizing levels, defined as induction levels below 15% of the mean value for controls tested the same day. The horizontal dotted lines represent Gyros auto-Abs levels **B**: Plot of anti–IFN-ω auto-Abs levels, as determined by Gyros, against their neutralization capacity at 100 pg/ml. The vertical dotted line indicates neutralizing levels, defined as induction levels below 15% of the mean value for controls tested the same day. The horizontal dotted lines represent Gyros auto-Abs levels

Lastly, no patients with neutralizing activity against 10 ng/ml of IFN-β were observed in our study. Overall, 7.9% of the patients display neutralizing activity against IFN-α and/or IFN-ω. Only 27% (3 of 11) of these patients with neutralizing auto-Abs presented high levels of auto-Abs in Gyros assay, other patients having intermediate levels in Gyros assay or even no detectable auto-Abs. Two patients with neutralizing auto-Abs against IFN-α and 3 patients with neutralizing auto-Abs against IFN-ω had no detectable auto-Abs in Gyros assay (Table 1). Moreover, unlike previous studies, where most samples with high titer of auto-Abs in Gyros assay were neutralizing *in vitro* [14], 79% (11 of 14) of patients with high levels of auto-Abs against IFN-α2 or IFN-ω in Gyros assay had no neutralizing activity *in vitro*. Of note, this was mostly observed for IFN-ω, where 100% of high titers were not neutralizing while 70% for IFN-α2 (Fig 1A and 1B).

### Increased mortality of patients with neutralizing auto-Abs to type I IFNs

We compared the clinical characteristics of patients having neutralizing auto-Abs against type I IFNs with patients without neutralizing auto-Abs (Table 2). Although differences were not significant with the patients without neutralizing auto-Abs, most of patients with neutralizing auto-Abs were men (82%). 82% of patients with neutralizing auto-Abs were over the age of 65 years and neutralizing auto-Abs were present in 12.5% of patients with critical COVID-19 over the age of 80 years.

In our study, 67% (93 of 139) of patients were labeled as having a full engagement status. The full engagement status was significantly less frequent in patient with auto-Abs (36%) than in patients without auto-Abs (69%). Nevertheless, no differences were seen between patients with or without auto-Abs for comorbidities and biological characteristics at hospital admission. Moreover, there was no difference in the proportion of patients that were transferred to an ICU between patients with or without auto-Abs.

Finally, it was recently shown that at least 18% of patients who died of COVID-19 pneumonia had auto-Abs capable of neutralizing 100 pg/ml type I IFNs in plasma 1/10 [14]. In our study, the mortality was significantly more frequent in patients with neutralizing auto-Abs as 55% (6 of 11) of patients with auto-Abs died versus 18% (23 of 128) of patients without auto-Abs. 21% of critical patients of our cohort who died from COVID-19 pneumonia had auto-Abs capable of neutralizing 100 pg/ml type I IFNs in plasma 1/10.

We compared clinic characteristics of deceased patients having neutralizing auto-Abs against type I IFNs with deceased patients without neutralizing auto-Abs. Although the deceased patients were usually elderly men, no significant differences were observed between the two groups (Table 3).

### Characteristics of patients with non neutralizing auto-Abs to type I IFNs

Many patients of the cohort had frequently auto-Abs, sometimes with high titer, without detected neutralizing activity. Indeed, 79% (11 of 14) of patients with high titer of auto-Abs had no apparent neutralizing activity *in vitro*.We compared characteristics of patients with neutralizing auto-Abs with patients with high titer auto-Abs without neutralizing activity and patients with intermediate titer auto-Abs without neutralizing activity (Table 4). Men and old patients were more frequent in patients with neutralizing Auto-Abs than in patients with auto-Abs without neutralizing activity but difference was not significant. Deaths were significantly more frequent in patients with neutralizing antibodies than in patients with intermediate titer auto-Abs without neutralizing activity (p=0.01). Moreover, there were more deaths in patients with neutralizing antibodies than in patients with high titer auto-Abs without neutralizing activity, without the difference being significant (p=0.18). These auto-Abs might be falsely positive or might be able to neutralize even lower amounts of type I IFNs.

### Detection of auto-Abs to type I IFNs by ELISA

All samples were tested for the presence of anti IFN-α auto-Abs by a commercially available ELISA kit assay. Interestingly, only 6.5% (9 of 139) of patients had anti IFN-α auto-Abs detectable by ELISA whereas 68% (94 of 139) patients have auto-Abs against IFN-α by Gyros technology.

Three patients had low levels of anti IFN-α auto-Abs (<100 ng/ml). These patients had also intermediate titer of anti IFN-α auto-Abs in Gyros assays, but no neutralizing activity.

**Fig 2:**
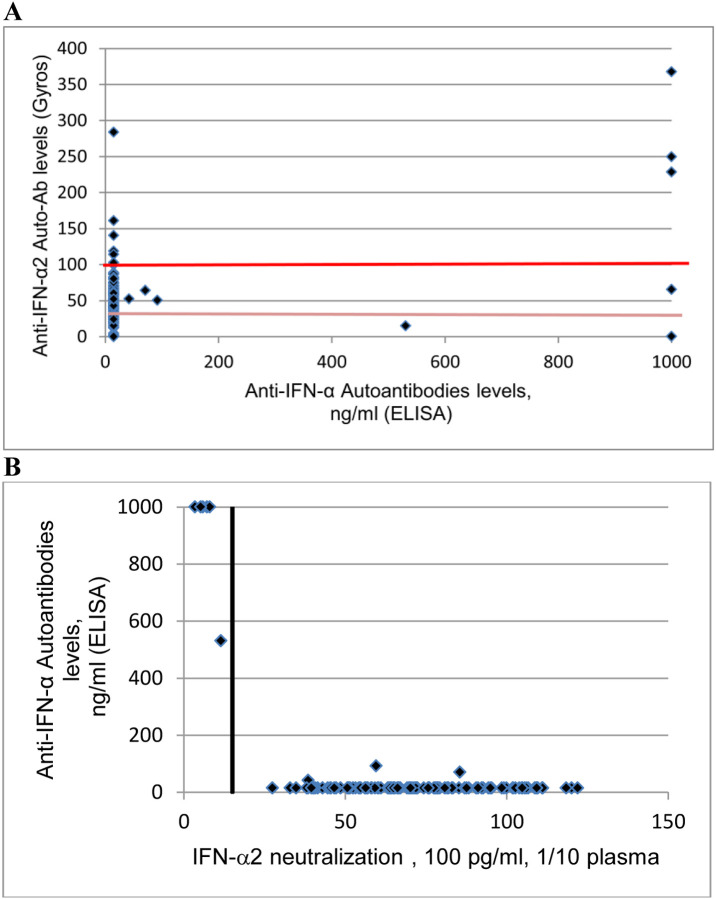
**A:** Plot of anti–IFN-α2 auto-Abs levels, as determined by Gyros, against anti–IFN-α2 auto-Abs levels, as determined by ELISA. The horizontal dotted lines represent Gyros auto-Abs levels **B** : Plot of anti–IFN-α2 auto-Abs levels, as determined by ELISA, against their neutralization capacity at 100 pg/ml. The vertical dotted line indicates neutralizing levels, defined as induction levels below 15% of the mean value for controls tested the same day

Moreover, six patients had high titer of anti IFN-α auto-Abs in ELISA (530.7 ng/ml or >1000 ng/ml). In Gyros assay, IFN-α auto-Abs were present with high levels in 3 patients, intermediate level in 1 patient and no dectectable in 2 patients (fig 2A).

Interestingly, all the 6 patients with high titer auto-Abs in ELISA have a neutralizing activity *in vitro* against IFN-α (Fig 2B). Three of these patients had auto-Abs neutralizing high concentrations of IFN-α2 and IFN-ω, 1 of these patients had auto-Abs neutralizing high concentrations of IFN-α2 and 2 of these patients had auto-Abs neutralizing lower concentrations of IFN-α2. High levels IFN-α auto-Abs were notably detected in ELISA in the 2 patients with neutralizing activity against IFN-α2 but without detectable auto-Abs in Gyros assay. Presence of high titer auto-Abs against IFN-α in ELISA seems to be better correlated with neutralizing activity of auto-Abs than Gyros technology.

**Fig 3:**
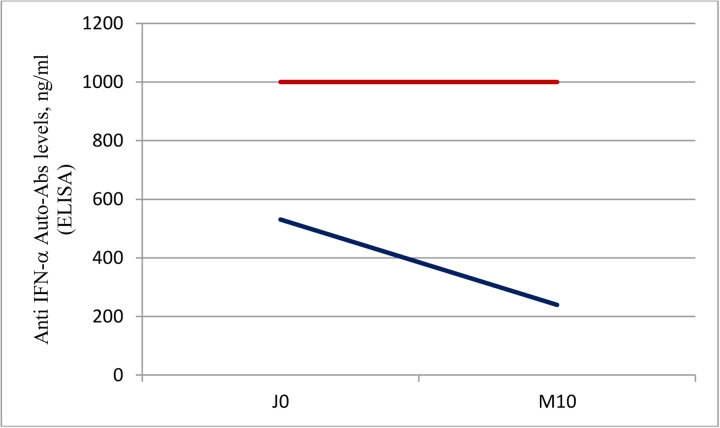
Detection of anti–IFN-α2 auto-Abs in patients 10 months after infection

Among the 6 patients with high titer IFN-α antibodies detected by ELISA, 3 patients survived. A sample was collected 10 months after COVID-19 pneumonia for 2 of these surviving patients and analyzed by ELISA. Interestingly, high levels of IFN-α antibodies were detected in these patients indicating a persistence of auto-Abs at least 10 months after COVID 19 infection (Fig 3). These patients continue to be followed in hospital, especially in order to see if they develop again a severe COVID 19 pneumonia or other viral infections.

## Discussion

We assessed the prevalence of auto-Abs against type I IFNs in 139 patients hospitalized for critical COVID-19 pneumonia in medicine departments dedicated to treat COVID-19 patients..

As many as 77% of patients with critical COVID-19 have auto-Abs against IFN-α and/or IFN-ω by Gyros technology, mainly with intermediate titer and less frequently with high titer. These results are much higher than those previously described as Bastard *et a*l described high or intermediate levels of IgG auto-Abs against IFN-α2 and/or IFN-ω in about 20% of patients with critical COVID-19 [14]. However, 79% of our patients with high titer of auto-Abs had no neutralizing activity. We found circulating auto-Abs that neutralized 100 pg/ml IFN-α2 and/or IFN-ω in plasma 1/10 in only 7.9% of our patients.

These results seem to indicate a lack of specificity of Gyros technology, especially for auto-Abs against IFN-ω and for intermediate titer of auto-Abs, or a lack of sensitivity of neutralization assays. Auto-Abs without neutralizing activity might be falsely positive or might be able to neutralize even lower amounts of type I IFNs.

We also evaluate a commercially available ELISA kit in our laboratory for detection of IFN-α auto-Abs. We did not search anti IFN-α auto-Abs by ELISA in general population. Nevertheless, according to manufacturer’s results, in a panel of 57 serum samples from randomly selected apparently healthy donors and patients suffering from various diseases, anti-IFN α levels ranged between 0 and 120.5 ng/ml (mean :17.4 ng/ml; SD : 26.7 ng/ml). Moreover, this commercial ELISA was recently evaluated [9]; a cut off of 34 ng/ml was established and results >100 ng/ml were considered as high. Three patients had anti IFN-α auto-Abs levels <100 ng/ml, which were considered as low, and also presented no neutralizing activity *in vitro* against IFN-α. In our study, 6 patients had high titer of anti IFN-α auto-Abs in ELISA and presented all a neutralizing activity *in vitro* against IFN-α. Goncalves *et al* [9] showed similar results and determined a cut-off of 1000 ng/ml correlated with neutralization assays. In their study, ability of auto-Abs to neutralize only high concentrations of IFN-α (10 ng/ml) was investigated. In our study, we tested the neutralizing activity of all these samples against IFNs *in vitro* at high concentrations and lower concentrations. In one of our patient, a concentration of 530.7 ng/ml was shown to be correlated with ability of auto-Abs to neutralize 100 pg/ml IFN-α2 in plasma 1/10 These results have to be confirmed in more samples and it is also necessary to determinate cut off correlated with neutralizing activity against low concentration of IFN-α. High levels IFN-α auto-Abs were notably detected in ELISA in 2 patients with neutralizing activity against IFN-α2 but without detectable auto-Abs in Gyros assay. The difference between Gyros technology and Invitrogen ELISA could be explained by the nature of the conjugate, secondary antibody, goat anti-human IgG and HRP–conjugated human IFN-α protein respectively, probably leading to a better detection of auto antibodies with ELISA. Overall, high titer auto-Abs against IFN-α in ELISA seems to be better correlated with neutralizing activity of auto-Abs than Gyros.

The proportion of patients with type I IFNs neutralizing auto-Abs is lower than those obtained in previous studies as it was described auto-Abs that neutralized 100 pg/ml IFN-α2 and/or IFN-ω in plasma 1/10 in 13.6% of the critical patients [14]. However, our cohort included only patients with critical COVID-19 hospitalized in medicine departments dedicated to treat COVID-19 patients, excluding patients with invasive mechanical ventilation and those in ICU before the admission in medecine. It was indeed notably constituted in order to assess associations between treatments and outcomes like need of ventilation mechanic and mortality [16]. Overall, severity of patients studied here was potentially lower than that of patients hospitalized in Intensive Care Units [5,6,9–11,14].

Previous studies showed that patients with neutralizing auto-Abs against type I IFNs were mostly men (94%) and that half were older than 65 years [5]. Moreover, it was recently shown that proportion of patients with critical COVID-19 having neutralizing auto-Abs increased with age [14]. In our study, although differences were not significant with the patients without neutralizing auto-Abs, most of patients (82%) with neutralizing auto-Abs were men and over the age of 65 years. The full engagement status was significantly less frequent in patient with auto-Abs than in patients without auto-Abs. but like other studies [7,9], there was no difference for comorbidities. Unlike study of Troya *et al* [7], where a significant correlation between presence of auto-Abs neutralizing type I IFNs, raised levels of C-Protein Reactive and low lymphocytes counts was observed, in our study, no difference was seen for these biological characteristics. It is nevertheless important to note that we compare biological characteristics obtained at hospital admission, and not maximum levels of C- Protein Reactive and minimum levels of lymphocytes count.

Neutralizing antibodies against type I INFs were described in almost all of patients with auto immune polyendocrinopathy syndrome type I [18–20] but also in patients with thymoma, myasthenia gravis [21] and systemic lupus erythematosus [22–24]. In our cohort, these autoimmune diseases were not described in patients with neutralizing auto-Abs, although 2 of 11 (18%) patients with neutralizing Auto-Abs against type I IFNs had a history of psoriasis. Research of antinuclear antibodies (ANA) did not reveal high titer of ANA in the 6 patients that were analyzed.

Like in a previous study [14], the presence of neutralizing auto-Abs is associated with a more frequent mortality. 21% of patients who died of COVID-19 pneumonia in our study had auto-Abs capable of neutralizing 100 pg/ml type I IFNs in plasma 1/10. Deceased patients with auto-Abs did not present overt clinical differences with deceased patients without auto-Abs.. These results confirms importance of the IFN-I pathway in the defense against SARS-CoV-2 infection.

Overall, the detection of neutralizing Auto-Abs against type I IFNs is therefore important due to clinical applications, notably therapeutics. Early identification of patients with auto-Abs should prompt early treatment and preventive management.

Lastly, our study describes persistence of neutralizing Auto-Abs almost one year after COVID-19 infection in two patients. Risk associated with this persistence is not known to date but must but explored, especially given the potential impact of these auto-Abs on the severity of other viral diseases, as described for adverse reactions following Yellow-fever vaccination [25].

## Figures and Tables

**Table 1:** Levels of AutoAbs obtained by Gyros in patients with neutralizing activity against type I IFNs.

	Neutralizing Auto-Abs	Gyros anti IFN-α	Gyros anti IFN-ω
Auto-Abs neutralizing only 10 ng/ml of type I IFNs			
Patient 1	Anti-IFN-α2 and anti IFN-ω	367,899**	25,908
Patient 2	Anti-IFN-α2 and anti IFN-ω	228,684**	30,122*
Patient 3	Anti-IFN-α2 and anti IFN-ω	249,683**	56,4271*
Patient 4	Anti-IFN-α2 only	0,395918	0,540107
Auto-Abs neutralizing only 100 pg/ml of type I IFNs			
Patient 5	Anti-IFN-α2 only	65,5719*	75,3769*
Patient 6	Anti-IFN-α2 only	15,1039	69,0513*
Patient 7	Anti-IFN-ω only	46,03*	55,9145*
Patient 8	Anti-IFN-ω only	52,352*	47,1288*
Patient 9	Anti-IFN-ω only	46,5971*	72,4223*
Patient 10	Anti-IFN-ω only	86,6252*	74,1435*
Patient 11	Anti-IFN-ω only	0,426105	0,333437

Levels of Auto-Abs against IFN-α and IFN-ω obtained by Gyros in the 11 patients with neutralizing activity are presented. Results are considered as negative if <30, positive with intermediate titer of Auto-Abs if >30 and <100 (*) and positive with high titer of Auto-Abs if >100 (**)

**Table 2:** Clinical characteristics of patients.

	Patients with neutralizing auto-Abs	Patients with auto-Abs without neutralizing activity or without antibodies	p values
Demographics			
n	11	128	
Age, mean ± Standard deviation (SD), years	68.7 ± 14.9	64 ± 15.8	p=0.29
≥ 65 years old	9 (82%)	65 (51%)	p =0.06
Sex (male)	9 (82%)	77 (60%)	p =0.2
Clinical features			
Full engagement	4 (36%)	89 (70%)	p=0.04
Diabetes	3 (27%)	51 (40%)	p =0.5
Obesity	3 (27%)	40 (31%)	p=1
HTA	4 (36%)	67 (52%)	p=0.4
History of cardiovascular disease, stroke, peripheral artery disease, heart failure	3 (27%)	27 (21%)	p=0.7
History of chronic obstructive pulmonary disease, asthma, emphysema, fibrosis	1 (9%)	22 (17%)	p=0.69
Solid organ transplantation	1 (9%)	0 (0%)	p=0.08
HIV	0 (0%)	2 (1,6%)	p =1
Immunosuppressant drugs and/or long-term oral corticosteroids	2 (18%)	10 (9%)	p =0.24
Malignancy (active)	2 (18%)	8 (6%)	p =0.18
Biological Characteristics at Hospital Admission			
C protein reactive, mean ± SD, mg/l	155 ± 89	124 ± 90	p=0.2
Lymphocyte count, mean ± SD, /μL	923 ± 269	1196 ± 1044	p=0.3
Creatinine mean ± SD, μmol/l	273 ± 434	84 ± 34	p=0;21
Clinical outcomes			
Intubed	1 (9%)	21 (16%)	p=1
Death	6 (55%)	23 (18%)	p=0.01

Data are presented as a number (percentage), unless otherwise noted. A Fisher test was used to analyze the effect of dichotomous variables and a Mann-Whitney test for continuous variables.

**Table 3:** Clinical characteristics of deceased patients.

	Deceased Patients with neutralizing auto-Abs	Deceased Patients with auto-Abs without neutralizing activity or without antibodies	p values
Demographics			
n	6	23	
Age, mean ± Standard deviation (SD), years	74.5 ± 12.6	75.3 ± 11.3	p=0.9
≥ 65 years old	5 (83%)	21 (87%)	p=1
Sex (male)	4 (67%)	15 (65%)	p=1
Clinical features			
Full engagement	1 (17%)	6 (26%)	p=1
Diabetes	2 (33%)	9 (39%)	p=1
Obesity	2 (33%)	5 (22%)	p=0.6
HTA	3 (50%)	15 (65%)	p=0.6
History of cardiovascular disease, stroke, peripheral artery disease, heart failure	1 (17%)	8 (35%)	p=0.6
History of chronic obstructive pulmonary disease, asthma, emphysema, fibrosis	0 (0%)	4 (17%)	p=0.5
Solid organ transplantation	1 (17%)	0 (0%)	p=0.2
Immunosuppressant drugs and/or long-term oral corticosteroids	2 (33%)	2 (9%)	p=0.2
Malignancy (active)	2 (33%)	2 (9%)	p=0.2
Clinical outcomes			
Intubed	1 (17%)	4 (17%)	p=1

Data are presented as a number (percentage), unless otherwise noted. A Fisher test was used to analyze the effect of dichotomous variables and a Mann-Whitney test for continuous variables.

**Table 4:** Clinical characteristics of with auto-Abs to type I IFNs detectable but non neutralizing

	Patients with intermediate titer of antibodies against type I IFNS without neutralizing activity	Patients with high titer of antibodies against type I IFNS without neutralizing activity	Patients with neutralizing auto-Abs against type I IFNs
n	87	11	11
Age, mean ± Standard deviation (SD), years	64.9 ± 16.2	60.5 ± 16.1	68.7 ± 14.9
≥ 65 years old	46 (53%)	5 (45%)	9 (82%)
men	51 (59%)	6 (55%)	9 (82%)
death	15 (17%)	2 (18%)	6 (55%)

Data are presented as a number (percentage), unless otherwise noted. A Fisher test was used to analyze the effect of dichotomous variables and a Mann-Whitney test for continuous variables

## Data Availability

Clinical data files are stored at Robert Ballanger Hospital. It may be shared if needed.

## References

[1] Levin A. T.; Hanage W. P.; Owusu-Boaitey N.; Cochran K. B.; Walsh S. P.; Meyerowitz-Katz G. Assessing the Age Specificity of Infection Fatality Rates for COVID-19: Systematic Review, Meta-Analysis, and Public Policy Implications. Eur. J. Epidemiol. 2020, 35 (12), 1123–1138. 10.1007/s10654-020-00698-1. 33289900 PMC7721859

[2] O’Driscoll M.; Ribeiro Dos Santos G.; Wang L.; Cummings D. A. T.; Azman A. S.; Paireau J.; Fontanet A.; Cauchemez S.; Salje H. Age-Specific Mortality and Immunity Patterns of SARS-CoV-2. Nature 2021, 590 (7844), 140–145. 10.1038/s41586-020-2918-0. 33137809

[3] Zhang Q.; Bastard P.; Liu Z.; Le Pen J.; Moncada-Velez M.; Chen J.; Ogishi M.; Sabli I. K. D.; Hodeib S.; Korol C.; Rosain J.; Bilguvar K.; Ye J.; Bolze A.; Bigio B.; Yang R.; Arias A. A.; Zhou Q.; Zhang Y.; Onodi F.; Korniotis S.; Karpf L.; Philippot Q.; Chbihi M.; Bonnet-Madin L.; Dorgham K.; Smith N.; Schneider W. M.; Razooky B. S.; Hoffmann H.-H.; Michailidis E.; Moens L.; Han J. E.; Lorenzo L.; Bizien L.; Meade P.; Neehus A.-L.; Ugurbil A. C.; Corneau A.; Kerner G.; Zhang P.; Rapaport F.; Seeleuthner Y.; Manry J.; Masson C.; Schmitt Y.; Schlüter A.; Le Voyer T.; Khan T.; Li J.; Fellay J.; Roussel L.; Shahrooei M.; Alosaimi M. F.; Mansouri D.; Al-Saud H.; Al-Mulla F.; Almourfi F.; Al-Muhsen S. Z.; Alsohime F.; Al Turki S.; Hasanato R.; van de Beek D.; Biondi A.; Bettini L. R.; D’Angio’ M.; Bonfanti P.; Imberti L.; Sottini A.; Paghera S.; Quiros-Roldan E.; Rossi C.; Oler A. J.; Tompkins M. F.; Alba C.; Vandernoot I.; Goffard J.-C.; Smits G.; Migeotte I.; Haerynck F.; Soler-Palacin P.; Martin-Nalda A.; Colobran R.; Morange P.-E.; Keles S.; Çölkesen F.; Ozcelik T.; Yasar K. K.; Senoglu S.; Karabela Ş. N.; Rodríguez-Gallego C.; Novelli G.; Hraiech S.; Tandjaoui-Lambiotte Y.; Duval X.; Laouénan C.; COVID Human Genetic Effort†; COVID-STORM Clinicians†; COVID Clinicians†; Imagine COVID Group†; French COVID Cohort Study Group†; CoV-Contact Cohort†; Amsterdam UMC Covid-19; Biobank†; NIAID-USUHS COVID Study Group†; Snow A. L.; Dalgard C. L.; Milner J. D.; Vinh D. C.; Mogensen T. H.; Marr N.; Spaan A. N.; Boisson B.; Boisson-Dupuis S.; Bustamante J.; Puel A.; Ciancanelli M. J.; Meyts I.; Maniatis T.; Soumelis V.; Amara A.; Nussenzweig M.; García-Sastre A.; Krammer F.; Pujol A.; Duffy D.; Lifton R. P.; Zhang S.-Y.; Gorochov G.; Béziat V.; Jouanguy E.; Sancho-Shimizu V.; Rice C. M.; Abel L.; Notarangelo L. D.; Cobat A.; Su H. C.; Casanova J.-L. Inborn Errors of Type I IFN Immunity in Patients with Life-Threatening COVID-19. Science 2020, 370 (6515), eabd4570. 10.1126/science.abd4570. 32972995 PMC7857407

[4] Asano T.; Boisson B.; Onodi F.; Matuozzo D.; Moncada-Velez M.; Maglorius Renkilaraj M. R. L.; Zhang P.; Meertens L.; Bolze A.; Materna M.; Korniotis S.; Gervais A.; Talouarn E.; Bigio B.; Seeleuthner Y.; Bilguvar K.; Zhang Y.; Neehus A.-L.; Ogishi M.; Pelham S. J.; Le Voyer T.; Rosain J.; Philippot Q.; Soler-Palacín P.; Colobran R.; Martin-Nalda A.; Rivière J. G.; Tandjaoui-Lambiotte Y.; Chaïbi K.; Shahrooei M.; Darazam I. A.; Olyaei N. A.; Mansouri D.; Hatipoğlu N.; Palabiyik F.; Ozcelik T.; Novelli G.; Novelli A.; Casari G.; Aiuti A.; Carrera P.; Bondesan S.; Barzaghi F.; Rovere-Querini P.; Tresoldi C.; Franco J. L.; Rojas J.; Reyes L. F.; Bustos I. G.; Arias A. A.; Morelle G.; Christèle K.; Troya J.; Planas-Serra L.; Schlüter A.; Gut M.; Pujol A.; Allende L. M.; Rodriguez-Gallego C.; Flores C.; Cabrera-Marante O.; Pleguezuelo D. E.; de Diego R. P.; Keles S.; Aytekin G.; Akcan O. M.; Bryceson Y. T.; Bergman P.; Brodin P.; Smole D.; Smith C. I. E.; Norlin A.-C.; Campbell T. M.; Covill L. E.; Hammarström L.; Pan-Hammarström Q.; Abolhassani H.; Mane S.; Marr N.; Ata M.; Al Ali F.; Khan T.; Spaan A. N.; Dalgard C. L.; Bonfanti P.; Biondi A.; Tubiana S.; Burdet C.; Nussbaum R.; Kahn-Kirby A.; Snow A. L.; Bustamante J.; Puel A.; Boisson-Dupuis S.; Zhang S.-Y.; Béziat V.; Lifton R. P.; Bastard P.; Notarangelo L. D.; Abel L.; Su H. C.; Jouanguy E.; Amara A.; Soumelis V.; Cobat A.; Zhang Q.; Casanova J.-L. X-Linked Recessive TLR7 Deficiency in ~1% of Men under 60 Years Old with Life-Threatening COVID-19. Sci. Immunol. 2021, 6 (62), eabl4348. 10.1126/sciimmunol.abl4348. 34413140 PMC8532080

[5] Bastard P.; Rosen L. B.; Zhang Q.; Michailidis E.; Hoffmann H.-H.; Zhang Y.; Dorgham K.; Philippot Q.; Rosain J.; Béziat V.; Manry J.; Shaw E.; Haljasmägi L.; Peterson P.; Lorenzo L.; Bizien L.; Trouillet-Assant S.; Dobbs K.; de Jesus A. A.; Belot A.; Kallaste A.; Catherinot E.; Tandjaoui-Lambiotte Y.; Le Pen J.; Kerner G.; Bigio B.; Seeleuthner Y.; Yang R.; Bolze A.; Spaan A. N.; Delmonte O. M.; Abers M. S.; Aiuti A.; Casari G.; Lampasona V.; Piemonti L.; Ciceri F.; Bilguvar K.; Lifton R. P.; Vasse M.; Smadja D. M.; Migaud M.; Hadjadj J.; Terrier B.; Duffy D.; Quintana-Murci L.; van de Beek D.; Roussel L.; Vinh D. C.; Tangye S. G.; Haerynck F.; Dalmau D.; Martinez-Picado J.; Brodin P.; Nussenzweig M. C.; Boisson-Dupuis S.; Rodríguez-Gallego C.; Vogt G.; Mogensen T. H.; Oler A. J.; Gu J.; Burbelo P. D.; Cohen J. I.; Biondi A.; Bettini L. R.; D’Angio M.; Bonfanti P.; Rossignol P.; Mayaux J.; Rieux-Laucat F.; Husebye E. S.; Fusco F.; Ursini M. V.; Imberti L.; Sottini A.; Paghera S.; Quiros-Roldan E.; Rossi C.; Castagnoli R.; Montagna D.; Licari A.; Marseglia G. L.; Duval X.; Ghosn J.; HGID Lab§; NIAID-USUHS Immune Response to COVID Group§; COVID Clinicians§; COVID-STORM Clinicians§; Imagine COVID Group§; French COVID Cohort Study Group§; The Milieu Intérieur Consortium§; CoV-Contact Cohort§; Amsterdam UMC Covid-19 Biobank§; COVID Human Genetic Effort§; Tsang J. S.; Goldbach-Mansky R.; Kisand K.; Lionakis M. S.; Puel A.; Zhang S.-Y.; Holland S. M.; Gorochov G.; Jouanguy E.; Rice C. M.; Cobat A.; Notarangelo L. D.; Abel L.; Su H. C.; Casanova J.-L. Autoantibodies against Type I IFNs in Patients with Life-Threatening COVID-19. Science 2020, 370 (6515), eabd4585. 10.1126/science.abd4585. 32972996 PMC7857397

[6] Koning R.; Bastard P.; Casanova J.-L.; Brouwer M. C.; van de Beek D.; with the Amsterdam U.M.C. COVID-19 Biobank Investigators; van Agtmael M.; Algera A. G.; Appelman B.; van Baarle F.; Bax D.; Beudel M.; Bogaard H. J.; Bomers M.; Bonta P.; Bos L.; Botta M.; de Brabander J.; Bree G.; de Bruin S.; Bugiani M.; Bulle E.; Chekrouni N.; Chouchane O.; Cloherty A.; Dongelmans D. A.; Dujardin R. W. G.; Elbers P.; Fleuren L.; Geerlings S.; Geijtenbeek T.; Girbes A.; Goorhuis B.; Grobusch M. P.; Hafkamp F.; Hagens L.; Hamann J.; Harris V.; Hemke R.; Hermans S. M.; Heunks L.; Hollmann M.; Horn J.; Hovius J. W.; de Jong M. D.; Koning R.; Lim E. H. T.; van Mourik N.; Nellen J.; Nossent E. J.; Olie S.; Paulus F.; Peters E.; van der Poll T.; Preckel B.; Prins J. M.; Raasveld J.; Reijnders T.; Schinkel M.; Schultz M. J.; Schuurmans A.; Schuurmans J.; Sigaloff K.; Slim M. A.; Smit M.; Stijnis C. S.; Stilma W.; Teunissen C.; Thoral P.; Tsonas A. M.; van der Valk M.; Veelo D.; de Vries H.; Vught L. A.; van Vugt M.; Wouters D.; Zwinderman A. H.; Brouwer M. C.; Wiersinga W. J.; Vlaar A. P. J.; van de Beek D. Autoantibodies against Type I Interferons Are Associated with Multi-Organ Failure in COVID-19 Patients. Intensive Care Med. 2021, 47 (6), 704–706. 10.1007/s00134-021-06392-4. 33835207 PMC8034036

[7] Troya J.; Bastard P.; Planas-Serra L.; Ryan P.; Ruiz M.; de Carranza M.; Torres J.; Martínez A.; Abel L.; Casanova J.-L.; Pujol A. Neutralizing Autoantibodies to Type I IFNs in >10% of Patients with Severe COVID-19 Pneumonia Hospitalized in Madrid, Spain. J. Clin. Immunol. 2021, 41 (5), 914–922. 10.1007/s10875-021-01036-0. 33851338 PMC8043439

[8] Vazquez S. E.; Bastard P.; Kelly K.; Gervais A.; Norris P. J.; Dumont L. J.; Casanova J.-L.; Anderson M. S.; DeRisi J. L. Neutralizing Autoantibodies to Type I Interferons in COVID-19 Convalescent Donor Plasma. J. Clin. Immunol. 2021, 41 (6), 1169–1171. 10.1007/s10875-021-01060-0. 34009544 PMC8132742

[9] Goncalves D.; Mezidi M.; Bastard P.; Perret M.; Saker K.; Fabien N.; Pescarmona R.; Lombard C.; Walzer T.; Casanova J.; Belot A.; Richard J.; Trouillet‐ Assant S. Antibodies against Type I Interferon: Detection and Association with Severe Clinical Outcome in COVID‐ 19 Patients. Clin. Transl. Immunol. 2021, 10 (8). 10.1002/cti2.1327. PMC8370568 34429968

[10] Wang E. Y.; Mao T.; Klein J.; Dai Y.; Huck J. D.; Liu F.; Zheng N. S.; Zhou T.; Israelow B.; Wong P.; Lucas C.; Silva J.; Oh J. E.; Song E.; Perotti E. S.; Fischer S.; Campbell M.; Fournier J. B.; Wyllie A. L.; Vogels C. B. F.; Ott I. M.; Kalinich C. C.; Petrone M. E.; Watkins A. E.; Yale IMPACT Team; Cruz C. D.; Farhadian S. F.; Schulz W. L.; Grubaugh N. D.; Ko A. I.; Iwasaki A.; Ring A. M. Diverse Functional Autoantibodies in Patients with COVID-19; preprint; Infectious Diseases (except HIV/AIDS), 2020. 10.1101/2020.12.10.20247205.

[11] van der Wijst M. G. P.; Vazquez S. E.; Hartoularos G. C.; Bastard P.; Grant T.; Bueno R.; Lee D. S.; Greenland J. R.; Sun Y.; Perez R.; Ogorodnikov A.; Ward A.; Mann S. A.; Lynch K. L.; Yun C.; Havlir D. V.; Chamie G.; Marquez C.; Greenhouse B.; Lionakis M. S.; Norris P. J.; Dumont L. J.; Kelly K.; Zhang P.; Zhang Q.; Gervais A.; Le Voyer T.; Whatley A.; Si Y.; Byrne A.; Combes A. J.; Rao A. A.; Song Y. S.; Fragiadakis G. K.; Kangelaris K.; Calfee C. S.; Erle D. J.; Hendrickson C.; Krummel M. F.; Woodruff P. G.; Langelier C. R.; Casanova J.-L.; Derisi J. L.; Anderson M. S.; Ye C. J.; on behalf of the UCSF COMET consortium. Type I Interferon Autoantibodies Are Associated with Systemic Immune Alterations in Patients with COVID-19. Sci. Transl. Med. 2021, eabh2624. 10.1126/scitranslmed.abh2624. 34429372 PMC8601717

[12] Lopez J.; Mommert M.; Mouton W.; Pizzorno A.; Brengel-Pesce K.; Mezidi M.; Villard M.; Lina B.; Richard J.-C.; Fassier J.-B.; Cheynet V.; Padey B.; Duliere V.; Julien T.; Paul S.; Bastard P.; Belot A.; Bal A.; Casanova J.-L.; Rosa-Calatrava M.; Morfin F.; Walzer T.; Trouillet-Assant S. Early Nasal Type I IFN Immunity against SARS-CoV-2 Is Compromised in Patients with Autoantibodies against Type I IFNs. J. Exp. Med. 2021, 218 (10), e20211211. 10.1084/jem.20211211. 34357402 PMC8352718

[13] Zhang Q.; Bastard P.; Bolze A.; Jouanguy E.; Zhang S.-Y.; Cobat A.; Notarangelo L. D.; Su H. C.; Abel L.; Casanova J.-L. Life-Threatening COVID-19: Defective Interferons Unleash Excessive Inflammation. Med 2020, 1 (1), 14–20. 10.1016/j.medj.2020.12.001. 33363283 PMC7748410

[14] Bastard P.; Gervais A.; Le Voyer T.; Rosain J.; Philippot Q.; Manry J.; Michailidis E.; Hoffmann H.-H.; Eto S.; Garcia-Prat M.; Bizien L.; Parra-Martínez A.; Yang R.; Haljasmägi L.; Migaud M.; Särekannu K.; Maslovskaja J.; de Prost N.; Tandjaoui-Lambiotte Y.; Luyt C.-E.; Amador-Borrero B.; Gaudet A.; Poissy J.; Morel P.; Richard P.; Cognasse F.; Troya J.; Trouillet-Assant S.; Belot A.; Saker K.; Garçon P.; Rivière J. G.; Lagier J.-C.; Gentile S.; Rosen L. B.; Shaw E.; Morio T.; Tanaka J.; Dalmau D.; Tharaux P.-L.; Sene D.; Stepanian A.; Megarbane B.; Triantafyllia V.; Fekkar A.; Heath J. R.; Franco J. L.; Anaya J.-M.; Solé-Violán J.; Imberti L.; Biondi A.; Bonfanti P.; Castagnoli R.; Delmonte O. M.; Zhang Y.; Snow A. L.; Holland S. M.; Biggs C.; Moncada-Vélez M.; Arias A. A.; Lorenzo L.; Boucherit S.; Coulibaly B.; Anglicheau D.; Planas A. M.; Haerynck F.; Duvlis S.; Nussbaum R. L.; Ozcelik T.; Keles S.; Bousfiha A. A.; El Bakkouri J.; Ramirez-Santana C.; Paul S.; Pan-Hammarström Q.; Hammarström L.; Dupont A.; Kurolap A.; Metz C. N.; Aiuti A.; Casari G.; Lampasona V.; Ciceri F.; Barreiros L. A.; Dominguez-Garrido E.; Vidigal M.; Zatz M.; van de Beek D.; Sahanic S.; Tancevski I.; Stepanovskyy Y.; Boyarchuk O.; Nukui Y.; Tsumura M.; Vidaur L.; Tangye S. G.; Burrel S.; Duffy D.; Quintana-Murci L.; Klocperk A.; Kann N. Y.; Shcherbina A.; Lau Y.-L.; Leung D.; Coulongeat M.; Marlet J.; Koning R.; Reyes L. F.; Chauvineau-Grenier A.; Venet F.; Monneret G.; Nussenzweig M. C.; Arrestier R.; Boudhabhay I.; Baris-Feldman H.; Hagin D.; Wauters J.; Meyts I.; Dyer A. H.; Kennelly S. P.; Bourke N. M.; Halwani R.; Sharif-Askari N. S.; Dorgham K.; Sallette J.; Sedkaoui S. M.; AlKhater S.; Rigo-Bonnin R.; Morandeira F.; Roussel L.; Vinh D. C.; Ostrowski S. R.; Condino-Neto A.; Prando C.; Bonradenko A.; Spaan A. N.; Gilardin L.; Fellay J.; Lyonnet S.; Bilguvar K.; Lifton R. P.; Mane S.; HGID Lab§; COVID Clinicians§; COVID-STORM Clinicians§; NIAID Immune Response to COVID Group§; NH-COVAIR Study Group§; Danish CHGE§; Danish Blood Donor Study§; St. James’s Hospital; SARS CoV2 Interest group§; French COVID Cohort Study Group§; Imagine COVID-Group§; The Milieu Intérieur Consortium§; CoV-Contact Cohort§; Amsterdam UMC Covid-19; Biobank Investigators§; COVID Human Genetic Effort§; CONSTANCES cohort§; 3C-Dijon Study§; Cerba Health-Care§; Etablissement du Sang study group§; Anderson M. S.; Boisson B.; Béziat V.; Zhang S.-Y.; Vandreakos E.; Hermine O.; Pujol A.; Peterson P.; Mogensen T. H.; Rowen L.; Mond J.; Debette S.; de Lamballerie X.; Duval X.; Mentré F.; Zins M.; Soler-Palacin P.; Colobran R.; Gorochov G.; Solanich X.; Susen S.; Martinez-Picado J.; Raoult D.; Vasse M.; Gregersen P. K.; Piemonti L.; Rodríguez-Gallego C.; Notarangelo L. D.; Su H. C.; Kisand K.; Okada S.; Puel A.; Jouanguy E.; Rice C. M.; Tiberghien P.; Zhang Q.; Cobat A.; Abel L.; Casanova J.-L. Autoantibodies Neutralizing Type I IFNs Are Present in ~ 4% of Uninfected Individuals over 70 Years Old and Account for ~ 20% of COVID-19 Deaths. Sci. Immunol. 2021, 6 (62), eabl4340. 10.1126/sciimmunol.abl4340. 34413139 PMC8521484

[15] Region L. P. La surmortalité durant l’épidémie de Covid-19 dans les départements franciliens https://www.ors-idf.org/nos-travaux/publications/la-surmortalite-durant-lepidemie-de-covid-19-dans-les-departements-franciliens/ (accessed 2021 −09 −09).

[16] Rossi B.; Nguyen L. S.; Zimmermann P.; Boucenna F.; Dubret L.; Baucher L.; Guillot H.; Bouldouyre M.-A.; Allenbach Y.; Salem J.-E.; Barsoum P.; Oufella A.; Gros H. Effect of Tocilizumab in Hospitalized Patients with Severe COVID-19 Pneumonia: A Case-Control Cohort Study. Pharmaceuticals 2020, 13 (10), 317. 10.3390/ph13100317. PMC7603074 33080877

[17] Clinical Spectrum https://www.covid19treatmentguidelines.nih.gov/overview/clinical-spectrum/ (accessed 2021 −09 −14).

[18] Meager A.; Visvalingam K.; Peterson P.; Möll K.; Murumägi A.; Krohn K.; Eskelin P.; Perheentupa J.; Husebye E.; Kadota Y.; Willcox N. Anti-Interferon Autoantibodies in Autoimmune Polyendocrinopathy Syndrome Type 1. PLoS Med. 2006, 3 (7), e289. 10.1371/journal.pmed.0030289. 16784312 PMC1475653

[19] Kärner J.; Meager A.; Laan M.; Maslovskaja J.; Pihlap M.; Remm A.; Juronen E.; Wolff A. S. B.; Husebye E. S.; Podkrajšek K. T.; Bratanic N.; Battelino T.; Willcox N.; Peterson P.; Kisand K. Anti-Cytokine Autoantibodies Suggest Pathogenetic Links with Autoimmune Regulator Deficiency in Humans and Mice: Anti-Cytokine Autoantibodies in Mice and Humans. Clin. Exp. Immunol. 2013, 171 (3), 263–272. 10.1111/cei.12024. 23379432 PMC3569533

[20] Bastard P.; Orlova E.; Sozaeva L.; Lévy R.; James A.; Schmitt M. M.; Ochoa S.; Kareva M.; Rodina Y.; Gervais A.; Le Voyer T.; Rosain J.; Philippot Q.; Neehus A.-L.; Shaw E.; Migaud M.; Bizien L.; Ekwall O.; Berg S.; Beccuti G.; Ghizzoni L.; Thiriez G.; Pavot A.; Goujard C.; Frémond M.-L.; Carter E.; Rothenbuhler A.; Linglart A.; Mignot B.; Comte A.; Cheikh N.; Hermine O.; Breivik L.; Husebye E. S.; Humbert S.; Rohrlich P.; Coaquette A.; Vuoto F.; Faure K.; Mahlaoui N.; Kotnik P.; Battelino T.; Trebušak Podkrajšek K.; Kisand K.; Ferré E. M. N.; DiMaggio T.; Rosen L. B.; Burbelo P. D.; McIntyre M.; Kann N. Y.; Shcherbina A.; Pavlova M.; Kolodkina A.; Holland S. M.; Zhang S.-Y.; Crow Y. J.; Notarangelo L. D.; Su H. C.; Abel L.; Anderson M. S.; Jouanguy E.; Neven B.; Puel A.; Casanova J.-L.; Lionakis M. S. Preexisting Autoantibodies to Type I IFNs Underlie Critical COVID-19 Pneumonia in Patients with APS-1. J. Exp. Med. 2021, 218 (7), e20210554. 10.1084/jem.20210554. 33890986 PMC8077172

[21] Meager A.; Wadhwa M.; Dilger P.; Bird C.; Thorpe R.; Newsom-Davis J.; Willcox N. Anti-Cytokine Autoantibodies in Autoimmunity: Preponderance of Neutralizing Autoantibodies against Interferon-Alpha, Interferon-Omega and Interleukin-12 in Patients with Thymoma and/or Myasthenia Gravis: Cytokine Autoantibodies, Thymoma and Myasthenia Gravis. Clin. Exp. Immunol. 2003, 132 (1), 128–136. 10.1046/j.1365-2249.2003.02113.x. 12653847 PMC1808678

[22] Howe H. S.; Leung B. P. L. Anti-Cytokine Autoantibodies in Systemic Lupus Erythematosus. Cells 2019, 9 (1), 72. 10.3390/cells9010072. PMC7016754 31892200

[23] Morimoto A. M.; Flesher D. T.; Yang J.; Wolslegel K.; Wang X.; Brady A.; Abbas A. R.; Quarmby V.; Wakshull E.; Richardson B.; Townsend M. J.; Behrens T. W. Association of Endogenous Anti-Interferon-α Autoantibodies with Decreased Interferon-Pathway and Disease Activity in Patients with Systemic Lupus Erythematosus. Arthritis Rheum. 2011, 63 (8), 2407–2415. 10.1002/art.30399. 21506093 PMC4028124

[24] Gupta S.; Tatouli I. P.; Rosen L. B.; Hasni S.; Alevizos I.; Manna Z. G.; Rivera J.; Jiang C.; Siegel R. M.; Holland S. M.; Moutsopoulos H. M.; Browne S. K. Distinct Functions of Autoantibodies Against Interferon in Systemic Lupus Erythematosus: A Comprehensive Analysis of Anticytokine Autoantibodies in Common Rheumatic Diseases: ANTI-INTERFERON FUNCTIONS IN SLE. Arthritis Rheumatol. 2016, 68 (7), 1677–1687. 10.1002/art.39607. 26815287 PMC5391258

[25] Bastard P.; Michailidis E.; Hoffmann H.-H.; Chbihi M.; Le Voyer T.; Rosain J.; Philippot Q.; Seeleuthner Y.; Gervais A.; Materna M.; de Oliveira P. M. N.; Maia M. de L. S.; Dinis Ano Bom A. P.; Azamor T.; Araújo da Conceição D.; Goudouris E.; Homma A.; Slesak G.; Schäfer J.; Pulendran B.; Miller J. D.; Huits R.; Yang R.; Rosen L. B.; Bizien L.; Lorenzo L.; Chrabieh M.; Erazo L. V.; Rozenberg F.; Jeljeli M. M.; Béziat V.; Holland S. M.; Cobat A.; Notarangelo L. D.; Su H. C.; Ahmed R.; Puel A.; Zhang S.-Y.; Abel L.; Seligman S. J.; Zhang Q.; MacDonald M. R.; Jouanguy E.; Rice C. M.; Casanova J.-L. Auto-Antibodies to Type I IFNs Can Underlie Adverse Reactions to Yellow Fever Live Attenuated Vaccine. J. Exp. Med. 2021, 218 (4), e20202486. 10.1084/jem.20202486. 33544838 PMC7871457

